# *Drosophilamelanogaster* Responses against Entomopathogenic Nematodes: Focus on Hemolymph Clots

**DOI:** 10.3390/insects11010062

**Published:** 2020-01-19

**Authors:** Alexis Dziedziech, Sai Shivankar, Ulrich Theopold

**Affiliations:** Department of Molecular Biosciences, The Wenner-Gren Institute (MBW), Stockholm University,10691 Stockholm, Sweden; alexis.dziedziech@su.se (A.D.); sai.krishnamoorthy@su.se (S.S.)

**Keywords:** insect immunity, innate immunity, hemocytes, secretion, nematodes, coagulation, clotting, phenoloxidase, transglutaminase

## Abstract

Several insect innate immune mechanisms are activated in response to infection by entomopathogenic nematodes (EPNs). In this review, we focus on the coagulation of hemolymph, which acts to stop bleeding after injury and prevent access of pathogens to the body cavity. After providing a general overview of invertebrate coagulation systems, we discuss recent findings in *Drosophila melanogaster* which demonstrate that clots protect against EPN infections. Detailed analysis at the cellular level provided insight into the kinetics of the secretion of *Drosophila* coagulation factors, including non-classical modes of secretion. Roughly, clot formation can be divided into a primary phase in which crosslinking of clot components depends on the activity of *Drosophila* transglutaminase and a secondary, phenoloxidase (PO)-dependent phase, characterized by further hardening and melanization of the clot matrix. These two phases appear to play distinct roles in two commonly used EPN infection models, namely *Heterorhabditis bacteriophora* and *Steinernema carpocapsae*. Finally, we discuss the implications of the coevolution between parasites such as EPNs and their hosts for the dynamics of coagulation factor evolution.

## 1. Introduction

### 1.1. Two Key Immune Pathways Are Dispensable in a Drosophila EPN Model

The life cycle of entomopathogenic nematodes (EPNs) requires that they breach epithelial barriers and create wounds in order to reach the hemolymph, where they complete their life cycle. Once inside their insect hosts, many EPN species release symbiotic pathogenic bacteria of the genera *Photorhabdus* or *Xenorhabdus* [1]. To varying extents, both the nematodes and their bacteria contribute to the success of EPN infections. Although EPN infections are known to induce immune factors, such as antimicrobial peptides, the two major immune pathways involved (imd and Toll) are shown to be dispensable for EPN control upon infection of *Drosophila melanogaster* with *Heterorhabditis bacteriophora/Photorhabdus luminescens* [2].

### 1.2. A Search for Alternative Immune Reactions against EPNs

Alternative potentially protective/immune responses against EPNs, which were identified in *Drosophila* and other insects comprise (see Box 1 for an overview of the reactions), (1) the formation of hemolymph clots at the wound site where nematodes enter (Box 1 and this review) and (2) encapsulation of nematodes [3,4,5]. The immune response of the host may target the symbiotic bacteria of the nematode through many potential mechanisms; however, the invading bacteria have often developed their own virulence factors which render the host’s response futile. Such responses on behalf of the host include (1) antimicrobial peptides [6,7], (2) phenoloxidase (PO) activity [8,9] (3) phagocytosis [10] and (4) nodulation i.e., the formation of aggregates, which contain immune effector cells (hemocytes [11]) and bacteria [12]. Encapsulation and nodule formation involve the release of extracellular components from hemocytes and lead to entrapment of larger objects, such as wasp eggs and nematodes (capsules) or larger numbers of bacteria (nodules). Encapsulation and nodule formation were functionally likened to the formation of granulomas in mammals [13]. At the cellular level, hemolymph coagulation, encapsulation and nodule formation show several similarities including extensive degranulation of hemocytes, the formation of an extracellular matrix and ultimately the conversion of prophenoloxidase (PPO) into its active form, PO [14]. PO (see also Box 1) is part of a multifunctional biochemical pathway with cytotoxic and crosslinking activity, which ultimately leads to the formation of melanin. Whether the similarities at the cellular level extend to the molecular mechanism remains to be determined. In this review, we summarize work on EPN infections in the model organism *Drosophila melanogaster* and a few additional insects, with focus on the coagulation- and the PPO-activating system. We also describe the *Drosophila* coagulation system in an evolutionary context in light of recent findings.

Box 1 ***Drosophila* immune responses [15].**

**Epithelial barriers:** epithelial barriers including the cuticle and the gut peritrophic membrane prevent most opportunistic organisms as well as many other microorganisms from gaining access to their insect hosts.

**Hemolymph coagulation/clotting:** at epithelial breaches, hemolymph components including soluble and hemocyte-derived factors form a matrix (clot) composed of a fibrous or gelatinous network, which seals the wound site. Clot components may be linked covalently (in *Drosophila* initially by transglutaminase and subsequently by phenoloxidase) or non-covalently (in many non-insect arthropods, see main text) and provide a substrate for further wound healing [16,17]. Coagulation helps to stop bleeding and to prevent entry of microbial intruders [18].

**Antimicrobial peptides (AMPs):** AMPs are expressed either constitutively or (in most cases) induced upon infection. They kill microbial intruders alone or in combination. AMPs as well as many molecules involved in other immune reactions are induced by two major pathways (imd and Toll) as well as through stress-related paths (JNK and JAK/STAT).

**Phagocytosis:** small microbial intruders are engulfed by a specialized class of hemocytes (plasmatocytes), which are akin to mammalian macrophages.

**Nodule formation:** although less studied in *Drosophila,* nodule formation in other insect is a reaction against larger numbers of bacteria, which exceed the phagocytic capacity of individual plasmatocytes. It leads to the formation of granuloma-like structures, which immobilize and kill bacteria.

**Encapsulation:** larger intruders, such as wasp eggs, are encapsulated initially by plasmatocytes and at later stages by lamellocytes. Lamellocytes are large flat cells, which are absent in naïve larvae and differentiate upon wasp infestation or wounding in hematopoietic organs or through trans-differentiation from plasmatocytes [19,20].

**The phenoloxidase-activating system (PAS):** the PAS involves a close collaboration between an extracellular proteolytic cascade, which is activated either by exogenous microbial elicitors or endogenous damage signals and hemocyte-derived prophenoloxidase (PPO). In *Drosophila*, two PPOs (PPO1 and PPO2) are harbored by a specialized class of hemocytes (crystal cells [21]), while a third one (PPO3) is produced by lamellocytes [22].

## 2. Hemolymph Coagulation in Non-Insect Arthropods

Due to their highly variable environments, coagulation systems in arthropods show a high level of variation [23] despite some coagulation factors (transglutaminase) and some coagulation factor domains (discoidin domain) being found across taxa [18]. Much of our knowledge of hemolymph coagulation stems from several model systems which were chosen due to their biochemical/genetic accessibility or because of their commercial interest. One such model is the horseshoe crab, an ancient arachnid in which coagulation involves activation of a serine protease cascade, known to be extremely sensitive to microbial elicitors such as lipopolysaccharides (LPS) from Gram-negative bacteria [24]. Ultimately, activation of the cascade leads to the cleavage of soluble coagulogen into insoluble coagulin, which forms non-covalently linked homopolymers [24,25]. Additional strengthening of the clot matrix occurs via transglutaminase (TG)-dependent crosslinking of proline-rich proteins (proxins, [26]). Proxins are also present on hemocyte surfaces, ensuring hemocyte incorporation into the clot [26]. All clot components in horseshoe crabs, including TG, are present in their hemocytes and are released upon stimulation by LPS [24,27]. This is in contrast to crustaceans, such as crayfish and lobster, in which TG that is derived from hemocytes or other tissues directly polymerizes plasma clottable proteins into clot fibers without the need for a proteolytic cascade [28,29]. Crayfish clottable protein shows no similarity to horseshoe crab coagulogen or mammalian fibrinogen but is instead related at the sequence level to insect vitellogenin with which it shares the presence of a von Willebrand factor D domain (vWF), which is common in mammalian coagulation factors [29,30]. In addition to TG-mediated coagulation, crayfish PPO is released from hemocytes and subsequently activated into phenoloxidase (PO) by a microbially induced proteolytic cascade [28,29]. Both TG and PPO lack classical signal peptides and are therefore released via non-classical secretion (NCS, [24,29]). In these arthropods, it is unknown whether TG and PPO are released simultaneously or at different time points during the coagulation reaction and whether this is mediated by different mechanisms of NCS.

## 3. Hemolymph Coagulation in Insects

Similar to crayfish, insect coagulation involves an interction between soluble hemolymph and hemocyte-derived components [15,18,31]. Earlier biochemical studies identified several hemolymph proteins in the clot, including the lipid carrier lipophorin and hemocytin, which shares several domains with vWF [31,32]. Invariably, most insect clots eventually melanize, indicating the presence of PO. Isolation of *Drosophila* clots confirmed the presence of lipophorin and the hemocytin homolog hemolectin in the clot matrix, as well as one of three *Drosophila* phenoloxidases (PPO2, [33]). Proteins newly identified as *Drosophila* coagulation factors using targeted proteomics and molecular genetics, included: the less-conserved fondue: a coagulation factor released from the fat body; tiggrin: a muscle attachment protein; Eig71eE: a salivary gland mucin [33,34]; hemomucin: a hemocyte surface protein [35]; glutactin: a basement membrane component produced by the fat body [36] and imaginal-disc growth factor 3, Idgf3: a protein with similarity to chitinase-like proteins (see Appendix A and text below [37]). Finally, a combination of inhibitor and genetic studies as well as bioinformatics showed that the secreted form of the only *Drosophila* TG member, a homolog of factor XIIIa, additionally contributes to clot formation [38]. The present view of *Drosophila* coagulation is that initially a clot forms upon wounding, although it is unclear whether this requires TG, as is the case in crayfish or whether TG acts on already existing (clotted) aggregates. However, it was shown that TG knockdown reduces clot formation and bacterial entrapment in flies, [38] as does factor XIIIa in humans [39]. After the clot seals the wound and captures bacteria preventing further dissemination, crystal cells [40] in the clot release PPO2 contributing additional bactericidal activity, further crosslinking of the clot matrix and eventually melanization [41,42]. In essence, clot formation in *Drosophila* larvae can be divided into an initial phase in which crosslinking depends on TG and a subsequent phase in which PO further crosslinks and hardens the clot. To fully appreciate the coordination between the two phases, it is essential to understand the release of the two key enzymes transglutaminase and PO from hemocytes and the recruitment and activation in clots to then further understand their mechanism of action against pathogenic threats.

## 4. Non-Classical Secretion (NCS) of *Drosophila* Coagulation Factors

Naïve *Drosophila* larvae contain two types of hemocytes: (1) plasmatocytes, which combine the activities of mammalian macrophages and granulocytes through their ability to phagocytose and release hemocyte coagulation factors and (2) crystal cells (CC). As their name indicates, crystal cells harbor several crystals, which contain PPO2. A second PO (PPO1) is located in the cytosol. The third type of hemocytes (lamellocytes) differentiate as a response towards larger intruders, such as wasp eggs, and upon wounding. Lamellocytes are crucial players during encapsulation and produce their own PO (PPO3). Recently, in order to understand two important proteins secreted using NCS, we used GFP-tagged versions of coagulation factors, namely PPO2 and TG. We showed that hemocyte-derived TG stays within the vicinity of hemocytes that are trapped in the clot, similar to what was found in horseshoe crab clots [24,41].

In contrast, ubiquitously expressed TG outlines the clot fibers, where it aids in hardening the clot matrix using fondue as substrate [34,38,42,43]. While hemocyte-TG had been previously shown to be targeted to recycling endosomes and released by exosomes [44,45], the mode of NCS remains to be determined for TG found in clot fibers but may involve cell rupture at wound sites or a form of compound secretion. Taken together, it appears that *Drosophila* TG combines the activity of crayfish and horseshoe crab TG by acting both as a clot crosslinker and by covalently trapping hemocytes in the clot matrix [41,42].

**Figure 1:**
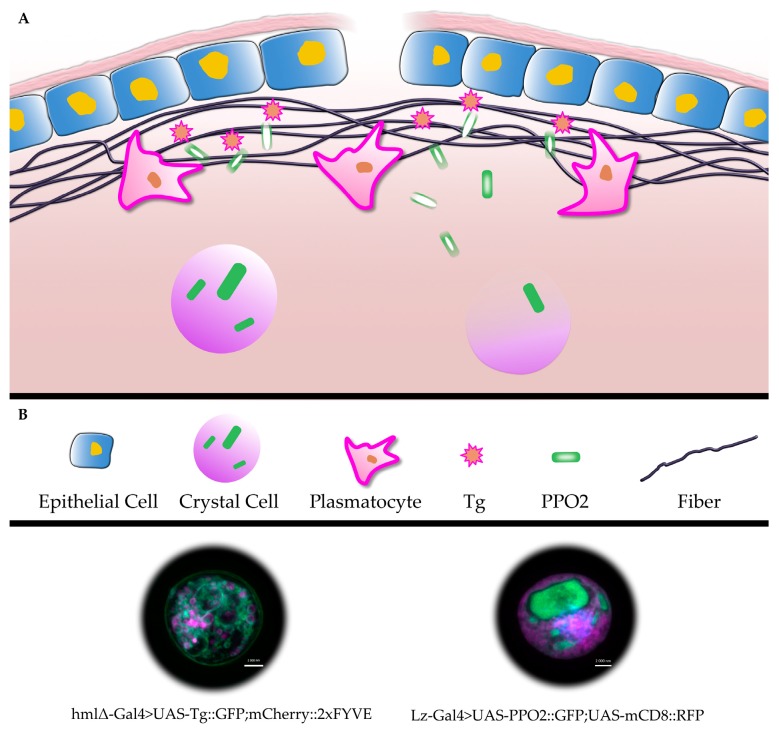
Schematic representation of clot formation in *Drosophila* larvae (**A**): structural components of the fibers (Fondue) are cross-linked, initially by transglutaminase and subsequently by phenoloxidase. (**B**) shows a key to the schematic as well as a plasmatocyte (lower part, inset to the left, with GFP-tagged transglutaminase) and a crystal cell (inset to the right, with GFP-tagged PPO2 in crystalline structures, nuclear staining with 4’,6-diamidino-2-phenylindole (DAPI), note that PPO2-containing crystals dissolve after release from crystal cells, indicated by a lighter shading [41] see text for further details).

In contrast to hemocyte-TG, PPO2 was found to be released through rupture of crystal cells which subsequently leads to the dissolution of its crystals within the clot where it acts both as a bactericide and as a crosslinker, in addition to TG [41]. This demonstrates that *Drosophila* hemocytes use two distinct modes of NCS for the release of TG and PPO2 respectively: exosomes for TG and cell rupture for PPO2 (see Figure 1B, plasmatocyte and crystal cell, lower panel). In addition, TG-dependent crosslinking precedes PPO2 activation, which requires CC rupture, dissolution of crystals and PPO activation [41]. These recent findings lead to a greater appreciation of NCS and the orchestration of coagulation factor secretion.

## 5. *Drosophila* Clots Protect against EPNs

**Figure 2:**
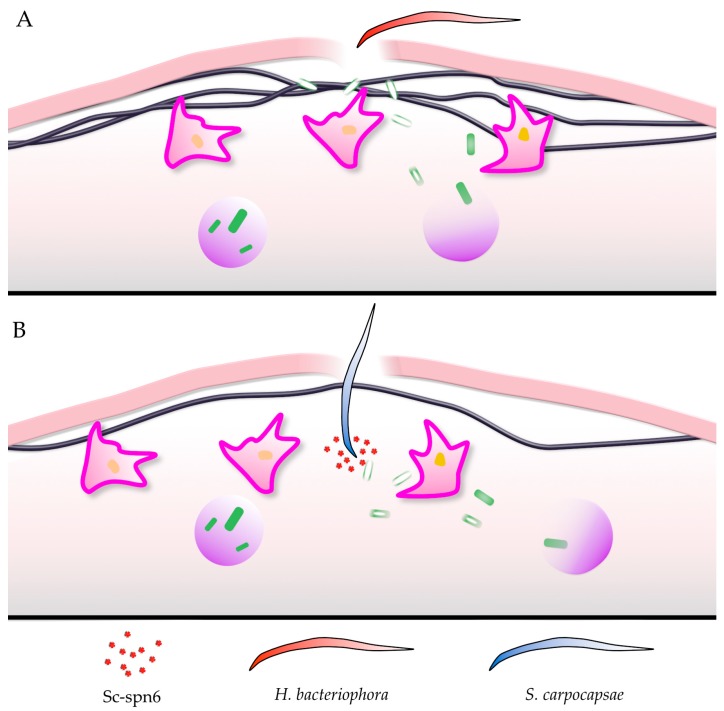
*Heterorhabditis bacteriophora* (**A**) and *Steinernema carpocapsae* (**B**) use different strategies to invade *Drosophila* larvae. Of key importance is that the *Steinernema* serpin Sc-spn6 prevents the association of phenoloxidase with the growing hemolymph clot, thereby reducing clot formation (see text for further details). The key indicates EPN species and Sc-spn6 found in *S. carpocapsae*.

When mutants or knockdown lines for coagulation factors were exposed to EPNs, some but not all were shown to have a protective effect (see Appendix A for details). When the combination *Heterorhabditis bacteriophora/Photorhabdus luminescens* was used, knockdowns and mutants for Fondue, glutactin, Idgf3, Eig71Ee and TG but not others (Hml, tiggrin, Hmu, Fbp1) displayed an increase in mortality [36,38,46]. Often the effects observed with EPNs were more significant than upon wounding [47] indicating that (1) coagulation shows some level of redundancy, for example by involving lipophorins/hexamerins and (2) some coagulation factors may be more relevant for immunity than for wound closure and healing [48] perhaps by modulating coagulation to protect against a given EPN. The latter aspect is strengthened by the fact that in addition to coagulation factors, a protective function of known immune molecules was found during EPN infections. These include immune recognition molecules such as a member of the Gram-negative binding protein family (GNPB3-like), a peptidoglycan receptor (PGRP-LF) and most notably, a member of the thioester-containing proteins, which are homologs of complement factors [36]. In mammals, a close collaboration between coagulation and complement was observed [49,50], and in insects, TEPs are known to intersect with different immune responses, including the PPO-activating system [51,52,53]. A role for all three *Drosophila* phenoloxidases was shown in the *Steinernema/Xenorhabdus* model (Table A1 in Appendix A) [9], while upon infection with *Heterorhabditis/Photorhabdus*, a mutant which lacks functional crystal cells (Black cell: Bc) and one that lacks an active PPO-activating protease (spn7) showed comparable mortality to controls (Table A1 and [38]). This indicates that even various aspects of clot formation may be of different importance depending on which EPN is studied (Figure 2A,B). In the case of *Steinernema carpocapsae,* a specific inhibitor (sc-spn6, Figure 2B [54]), inhibits PPO recruitment to the clot and the clot’s maturation, rendering the clot ineffective at preventing nematode entry. As a consequence of failed recruitment to the clot, PO activity in the presence of sc-spn6 is instead enriched in the hemolymph. We hypothesize that upon infection with *S. carpocapsae*, PO activity contributes to the host response in the hemolymph after nematode entry, while PO’s contribution to the response against *H. bacteriophora* is restricted to the clot.

## 6. Other Immune Factors—Eicosanoids as Mediators of Anti-EPN Responses

An essential biochemical class of immune mediators with relevance during EPN infections are eicosanoids [55]. In mammals, the first step in the production of eicosanoids involves the release of lipids from cell membranes by phospholipase A2 (PLA2). Symbiotic bacteria from EPN complexes, *Photorhabdus* and *Xenorhabdus,* were shown to inhibit PLA2 leading to immune suppression in lepidopteran insect hosts [55,56,57]. Supporting the importance of insect eicosanoids during EPN infections, an effect of knocking down a *Drosophila* PLA2 was shown [46]. The remaining part of the synthetic pathway remained elusive since using the Basic Local Alignment Search Tool prevailed no obvious homologs for cyclooxygenases or lipoxygenases that could be identified in insect genomes. Still, biochemical evidence pointed towards related paths [46,55] and more recently, advanced bioinformatics and genetic evidence identified novel genes involved in eicosanoid production and novel key lipid mediators in *Drosophila* and the mosquito, *Anopheles gambiae* [58,59,60]. Functional analysis confirmed the genes’ involvement in regulating inflammatory reactions in *Drosophila* as well as in hemocyte recruitment and immune priming upon *Plasmodium* infection in *Anopheles*. These genes and their *Drosophila* homologs will be interesting to evaluate in EPN models, particularly in regard to their effects on host hemocytes, clot formation and their potential for immune priming.

## 7. Moving beyond Insects—How Evolution Shapes Clot Formation

Although insects with their open circulatory system have a much-reduced risk of thrombus formation—one of the important adverse side effects of coagulation—clot formation in insects is still tightly regulated and limited to the proximity of wounds and nematode entry sites, leading to a compartmentalization of the hemolymph. This division between the open circulatory system and the coagulation region allows hemocytes to be trapped in the clot matrix while preventing their activation in other localities. Similarly, antibacterial (lysozyme) and PO activity are enriched at wound sites [61]. In addition, PO activity is more durable when microbial elicitors are co-injected at wound sites [61], an observation in line with the dual function of clots during hemostasis and immunity. The relative importance of microbial elicitors (microbe-associated molecular patterns, MAMPs) versus internal signals (damage-associated molecular patterns DAMPs, [62]) may differ between insects. For example, clots from mosquitoes and flies show a substantially more localized pattern of melanization, often associated with cell fragments in comparison to a more diffuse pattern in clots from two lepidopteran species [63,64]. One damage-associated mechanism and a possible explanation for localized melanization is the release of microparticles from hemocytes which—similar to platelet microparticles—are pinched off from hemocyte filopodia and therefore expose negatively charged phospholipids (phosphatidylserine, PS) on their surface [32,65]. Platelet PS increases the activity of coagulation factors, which assemble on the surface of microparticles. In *Drosophila*, externally added PS acts as a more potent activator of PO activity than microbial elicitors and may thus have an equivalent function to mammalian PS, which confers increased procoagulant activity to microparticle-bound coagulation factors [66].

Hemolymph coagulation and blood coagulation thus serve to both seal wounds and—at least in many cases—prevent dissemination of microbial intruders from the wound site. One central coagulation factor that is functionally conserved in both flies and humans is TG (factor XIIIa in humans), which serves to crosslink clot proteins and to immobilize bacteria in the clot [38,39,67]. We propose that in the *Heterorhabditis/Photorhabdus* system, the primary protective effect of coagulation lies in preventing nematode entry in the first place. The clot’s protective effect is circumvented by other EPNs where effective coagulation is inhibited, for example in *Steinernema carpocapsae* where sc-spn6 [54] inhibits recruitment of PO to the clot and thus proper clot maturation. In this way, EPN-host interactions are part of a classical evolutionary race, where the EPNs target different aspects of host behavior, physiology and immunity, and the insect hosts, in turn, adapt via different anti-parasitic pathways. One such example is the balance between the initial phases of coagulation and the subsequent melanization [54,68]. If one accepts the clot’s dual function during hemostasis and immunity, a hypercoagulatory phenotype may provide an evolutionary advantage during phases of increased risk of injury or infection via epithelial surfaces. In mammals, the evolutionary advantages of a hypercoagulatory phenotype have to be balanced against the risk of tissue damage during sepsis and microvascular thrombosis, in other words, there are trade-offs between both functions [69]. The balance between a hemostatic and an immune role will be influenced by different environments and by changes in the environment during evolution both at the macro- and microevolutionary scale. For example, it was proposed that introgression of a hypercoagulatory phenotype from Neanderthal genomes into human genomes may have conferred improved protection against wounds/infections, which in an urban environment is outweighed by an increased risk of cardiovascular diseases [70]. Similarly, despite the protective effect of insect PO against some EPNs, tissue damage is often a negative consequence of PO activity and it therefore must be tightly controlled [31,32]. In addition to the physical environment, coevolution with potential parasites will shape PO-activation and coagulation systems in insects and mammals, respectively. For both groups, an evolutionary perspective may thus help us to appreciate the darker side of hemostatic defects as a protective convergently evolved trade-off response against intruders, such as nematodes [69,70].

## Appendix A

**Table A1:** *Drosophila* genes tested for their effects upon infection with EPNs (+: effects were observed; -: no effects; Fb: effects upon knockdown in fat body; Hc: effects upon knockdown in hemocytes).

Gene	Effect on EPNs	EPN Used	References
imd	-	*Heterorhabditis/Photorhabdus*	[2]
Tl	-	*Heterorhabditis/Photorhabdus*	[2]
Transglutaminase	+	*Heterorhabditis/Photorhabdus*	[38]
Tiggrin	-	*Heterorhabditis/Photorhabdus*	[46]
Fondue	+ (Fb)	*Heterorhabditis/Photorhabdus*	[46]
Eig71Ee	+ (Hc)	*Heterorhabditis/Photorhabdus*	[46]
Fbp1	-	*Heterorhabditis/Photorhabdus*	[46]
Hemomucin	-	*Heterorhabditis/Photorhabdus*	[46]
Hemolectin	-	*Heterorhabditis/Photorhabdus*	[38]
GNBP-like 3	+ (Fb)	*Heterorhabditis/Photorhabdus*	[36]
PGRP-LF	+	*Heterorhabditis/Photorhabdus*	[36]
TEP3	+	*Heterorhabditis/Photorhabdus*	[36]
Glutactin	+ (Hc)	*Heterorhabditis/Photorhabdus*	[36]
Idgf3	+	*Heterorhabditis/Photorhabdus*	[37]
CG 14507 (PLA2)	+	*Heterorhabditis/Photorhabdus*	[46]
PPO1	+	*Steinernema/Xenorhabdus*	[9]
PPO2	+	*Steinernema/Xenorhabdus*	[9]
PPO3	+	*Steinernema/Xenorhabdus*	[9]
Bc	-	*Heterorhabditis/Photorhabdus*	[38]
Sp7 (PPO activation)	-	*Heterorhabditis/Photorhabdus*	[38]
